# Immobilization of a Laccase/2,2'-azino-bis-(3-ethylbenzothiazoline)-6-sulfonic Acid System to Layered Double Hydroxide/Alginate Biohybrid Beads for Biodegradation of Malachite Green Dye

**DOI:** 10.1155/2018/5471961

**Published:** 2018-09-24

**Authors:** Juan Huang, Yun Yang, Yaokun Wang, Mingyang Zhang, Youxun Liu

**Affiliations:** ^1^School of Life Sciences and Technology, Xinxiang Medical University, Jinsui Avenue 601, Xinxiang, Henan 453003, China; ^2^School of Basic Medical Sciences, Xinxiang Medical University, Jinsui Avenue 601, Xinxiang, Henan 453003, China; ^3^Key Laboratory of molecular medicine of Xinxiang, Jinsui Avenue 601, Xinxiang, Henan 453003, China

## Abstract

The application of laccase-mediator-based catalysis is limited owing to the high cost of laccases and mediators and the potential toxicity of free mediators. Here, a novel biocatalyst (Im-LMS) was fabricated by immobilizing both laccase and a mediator (2,2'-azino-bis-[3-ethylbenzothiazoline]-6-sulfonic acid) on layered double hydroxide/alginate biohybrid beads. The catalytic activity of Im-LMS was evaluated for dye decolorization using malachite green. The decolorization yields of malachite green by Im-LMS and the free laccase-mediator system were 92% within 120 min and 90% within 90 min. Malachite green solution was detoxified completely after biodegradation by Im-LMS. Following eight reuse cycles of Im-LMS for dye treatment, a decolorization yield of 79% was obtained. The activity of Im-LMS was almost completely stable after being stored for 10 days. The recyclability and stability of Im-LMS will be helpful for reducing the running cost and potential toxicity associated with mediators to facilitate practical applications.

## 1. Introduction

Laccases (polyphenoloxidase, EC 1.10.3.2) are a group of oxidases containing four copper atoms in the active site and are widespread in specific higher plants, fungi, and bacteria [1]. These enzymes have special abilities to oxidize a wide range of organic substances such as phenols, polyphenols, and anilines, with a simultaneous four-electron reduction of oxygen to water [2–4]. Furthermore, in the presence of appropriate small redox mediators, such as 1-hydroxybenzotriazole (HOBT) and 2,2'-azino-bis-(3-ethylbenzothiazoline)-6-sulfonic acid (ABTS), the range of substrates for laccase can be extended to nonphenolic compounds or compounds that are more difficult to oxidize [5]. Once oxidized by laccase, the mediator can diffuse away from the enzymatic pocket and successively oxidize other molecules via a nonenzymatic reaction with the reduction of the oxidized mediator, and the reduced mediator can then enter into the next catalytic cycle [6]. The laccase-mediator system (LMS) has been shown to efficiently oxidize various compounds [7]. Thus, LMSs have considerable potential for various biotechnological applications, such as paper pulp bleaching, organic synthesis, biofuel cells, and bioremediation [8].

However, the major limiting factors of free enzyme and dissolved mediators in solution for large-scale application are low stability and reusability and high production cost. Immobilization is a potential technique that has been explored to overcome these limitations. Various methods for laccase immobilization can be found in the literature [9, 10]. The enzyme can be immobilized onto various carriers and supports or entrapped within the capsule, markedly improving stability, enabling their reuse, and lowering the process cost. Moreover, some investigators have attempted to immobilize mediators in order to recover and reuse them. For example, Mendoza and coworkers have confirmed that the mediator 2,2,6,6-tetramethylpiperidine 1-oxyl (TEMPO) coupled to a poly(ethylene glycol) (PEG) molecule can be reused for azo dye decolorization by laccase [11]. Silica nanoparticles modified with the mediator ABTS have been successfully used for laccase-catalyzed dye decolorization[12]. Furthermore, ABTS immobilized on Metal-Organic Framework MIL-100(Fe) has been shown to be an efficient mediator for laccase-catalyzed decolorization [13]. Although the immobilization of the enzyme or mediator alone has been studied, few reports have described the coimmobilization of laccase and mediator, and studies examining the possibility of reusing LMS are still insufficient [14]. Free LMSs have been used for decolorization of a wide variety of dyes, and the results are highly promising [8, 15]. Moreover, the immobilization of LMSs has many important advantages, such as reusability, ease of use and low cost in practical applications for dye decolorization.

Layered double hydroxides (LDHs) are two-dimensional layered inorganic solids showing characteristics of anionic clays [16]. Their versatile features, such as tunable surface and porosity properties, unique anion exchange capacities, and adjustable layer charge density, has attracted increasing attention [17]. LDH materials can be designed as host matrices for immobilization of biomolecules, including amino acids, DNA, and enzymes, and intercalation of various organic molecules, such as ABTS, nitroxide, and porphyrins [18]. Furthermore, the opened structures of LDH offer favorable environments for interactions with enzymes and for redox mediator intercalation [19]. Horseradish peroxidase has been successfully immobilized into redox-active LDH to construct an electrochemical biosensor [20]. Intercalated redox mediators, including ABTS in LDH, are believed to play a major role in electron transfer between the electrode and the redox protein, resulting in improvement of the electrochemical transduction step.

**Scheme 1:**
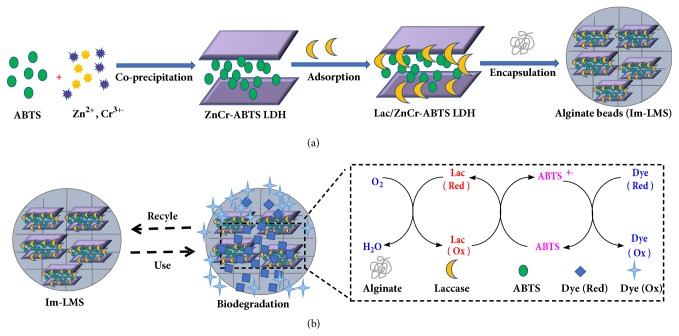
Schematic illustration of the strategy for preparation of Lac/ZnCr-ABTS LDH/alginate beads and MG biodegradation.

In the present paper, we propose a simple procedure for the noncovalent coimmobilization of laccase and ABTS. The main objective was to evaluate whether this immobilization of LMS was a practical strategy for the reuse of both laccase and its mediator for dye decolorization. Malachite green (MG), a representative type of toxic triarylmethane dye, was used as a model pollutant. As illustrated in Scheme 1, in the first step, ABTS was intercalated within LDH materials by the direct coprecipitation method. Subsequently, the laccase was adsorbed onto the surface of ABTS-hybrid LDH. Second, the hybrids were encapsulated in alginate beads in order to avoid leakage of the enzyme and mediator, leading to coimmobilization of laccase and ABTS. Third, the catalytic activity, recyclability, and stability of the biohybrid beads were investigated. Finally, the degradation products of MG were studied using an ultraviolet-visible (UV-Vis) spectrophotometer and matrix-assisted laser desorption/ionization time-of-flight mass spectrometry (MALDI-TOF-MS). In addition, the toxicities of the treated and untreated MG were assessed using antimicrobial activity tests and genotoxicity assays.

## 2. Materials and Methods

### 2.1. Materials

Laccase (EC 1.10.3.2, enzyme activity ≥ 0.6 U/mg) was provided by Sunson Industry Group (Beijing, China), produced by genetically modified microorganisms (*Aspergillus oryzae*). ABTS was supplied by Sigma-Aldrich (St. Louis, MO, USA). MG, zinc chloride (ZnCl_2_), and chromium chloride (CrCl_3_·6H_2_O) were purchased from Aladdin Bio-Chem Technology (Shanghai, China).* Escherichia coli* CICC 23872 and* Staphylococcus aureus* CICC 23926 were obtained from China Center of Industrial Culture Collection. HepG2 cells were a gift from Professor Changzheng Li (Xinxiang Medical University, China). All other chemicals and reagents were of analytical grade.

### 2.2. Preparation of ZnCr LDH, ZnCr-ABTS LDH, and Lac/ZnCr-ABTS LDH/Alginate Beads

The synthesis of ZnCr LDH and ZnCr-ABTS LDH was carried out by the coprecipitation method, as described previously [21]. The fabrication of Lac/ZnCr-ABTS LDH/alginate beads was performed as follows. Typically, 1 mL laccase solution (20 U) was added to a centrifuge tube containing 50 mg ZnCr-ABTS LDH dispersed in 1 mL of water. The above mixtures were shaken at 100 rpm for 2 h to ensure complete adsorption and then added to 9 mL sodium alginate solution (3.0% w/v) with stirring. The mixtures were dropped into 0.2 M CuSO_4_ crosslinker solutions using a syringe with a needle (18 G). After 1 h, the obtained beads were washed several times with distilled water to remove reagent excess. The synthesized Lac/ZnCr-ABTS LDH/alginate beads were denoted as Im-LMS. The immobilization yield was determined as residual laccase activity found after dissolution of beads compared with the laccase activity added to the alginate solution. Free and immobilized laccase activities were determined spectrophotometrically, as described previously [14].

### 2.3. Dye Decolorization

MG dye was used as the model pollutant for enzymatic decolorization. The effects of pH on dye decolorization by the free LMS and Im-LMS were examined between pH 4.0 and 6.5 (in 0.05M acetate buffer). Typically, the reaction mixture contained 20 mg/L dye, 30 mg Im-LMS (wet weight), and 4.0 mL acetate buffer (pH 6.0), and the mixture was incubated in a 5 mL centrifuge tube and shaken at 100 rpm at 25°C for 2 h. The extent of MG decolorization was determined spectrophotometrically based on a decrease in the absorbance of the dye at *λ* = 618 nm. Dye decolorization (%) was calculated according to the following formula: decolorization (%) = (A0 – At) / A0, where A0 is the initial absorbance of the dye solution at *λ* = 618 nm, and At is the absorbance of the dye solution after the desired reaction time. The capacity of Im-LMS for repeated decolorization was evaluated over eight cycles. After each reaction cycle, Im-LMS was recovered, washed three times with deionized water, and then fed into a new cycle. The experiments were performed at least in triplicate. The data shown in all figures correspond to mean values with standard errors.

### 2.4. Electrochemical Analysis

Electrochemical experiments were carried out using a CHI660A electrochemical workstation with a conventional three-electrode system. A glassy carbon (GC) electrode with a diameter of 4 mm was used as the working electrode, a saturated calomel electrode was used as the reference electrode, and a platinum electrode was employed as the auxiliary electrode. The ZnCr LDH or ZnCr-ABTS LDH-modified GC electrode was prepared as follows. First, 100 *μ*L ZnCr LDH or ZnCr-ABTS LDH (1 mg/mL) was mixed with 50 *μ*L of 1.0 wt% Nafion solution, and 20 *μ*L of this mixture was placed on the surface of the GC electrode to prepare a thin layer, which was allowed to dry at 50°Cfor 2 h. The acetate buffer solution (pH 6.0) was used as the supporting electrolyte. All cyclic voltammetric (CV) experiments were carried out at 25°C.

### 2.5. Stability Analysis

Considering ABTS mediator and laccase are the two major factors that affect the decolorization capacity of Im-LMS, the storage stability of Im-LMS in this paper refers to the possible leaching of mediator ABTS and the loss of laccase activity in Im-LMS during storage. The storage stabilities of the free laccase and Im-LMS were evaluated as follows. The free laccase and Im-LMS were stored at 4°C in water, and the laccase activities were determined periodically over a total duration of 10 days. The initial laccase activities were set as 100%, and the relative activities were defined as the ratio of the initial activities. To evaluate the possible leakage of ABTS from Im-LMS, ABTS was detected in the supernatant solution when Im-LMS samples were stored in water at 4°C. Changes in the absorbance spectrum of ABTS were monitored by UV-Vis spectroscopy at different time intervals.

### 2.6. Dye Biodegradation

The degradation of MG by Im-LMS was carried out in pH 6.0 acetate solution at 25°C. The reaction mixture was incubated in a centrifuge tube wrapped with aluminum foil to avoid photodegradation. At different time intervals, the supernatant from the reaction mixture was recovered and filtered using a 0.22 *μ*m membrane for degradation analysis. In order to monitor the extent of MG degradation, changes in the absorbance spectra of the dye were recorded in the UV-visible range between 200 and 800 nm. After complete decolorization of MG, the supernatant was analyzed by MALDI-TOF-MS to identify the degradation products as previously described [22].

### 2.7. Toxicity Analysis

The toxicities of MG and its degradation products were examined using antimicrobial activity tests and genotoxicity assays. Antimicrobial activity tests were carried out using* E. coli* CICC 23872 and* S. aureus *CICC 2392 as standard organisms. All strains were precultured in 5 mL LB broth at 37°C overnight. Approximately 0.15 mL liquid culture of each bacterial strain (OD_600_ = 0.5) was swabbed on the surface of LB agar plates. The paper disks were soaked with MG or its degradation products and then placed onto LB agar plates. Subsequently, the plates were incubated at 37°C for 24 h. Antimicrobial activity tests were then performed by measuring the diameters of the inhibition zones on plates. Genotoxicity assays were determined using comet assays, as previously described [23]. The statistical significance of differences was compared and analyzed using Student's t-tests or one-way analysis of variance. Differences with *P* values of less than 0.05 were considered statistically significant.

### 2.8. Characterization Studies

Fourier transform-infrared (FT-IR) spectroscopy was performed with a Tensor 27 spectrometer (Bruker, Germany) with the KBr pellet technique. Field emission scanning electron microscopy (SEM) and energy dispersive spectrometry (EDS) were performed with a JSM 6700F (JEOL, Japan). UV-Vis spectroscopy was performed with a Varian CARY50 spectrophotometer (USA). Transmission electron microscopy (TEM) was performed using a model 9000 TEM (Hitachi, Japan). CV measurements were carried out using a CHI600E electrochemical workstation (CH Instruments). Thermogravi-metric analysis (TGA) of the as-synthesized LDH matrix was performed with a LENSES STAPT-1000 calorimeter (Germany) by scanning up to 700°C with a heating rate of 10°C/min. Finally, ultrahigh resolution mass spectra were acquired using a solariX MALDI-TOF/TOF MS spectrometer (Bruker) in positive-ion mode.

## 3. Results and Discussion

### 3.1. Synthesis and Characterization of LDH Matrix

**Figure 1:**
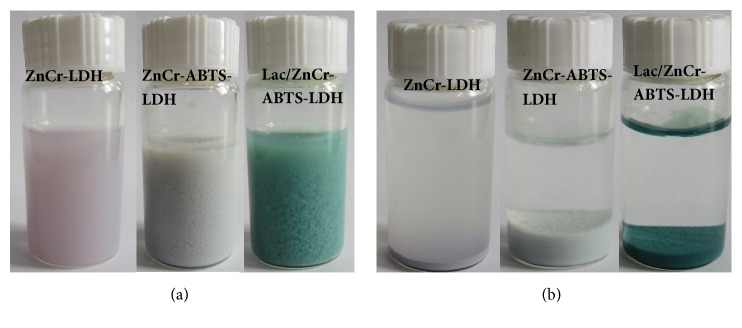
Photographic images of** (a)** suspensions and** (b)** precipitates of the as-synthesized LDH matrix.

**Figure 2:**
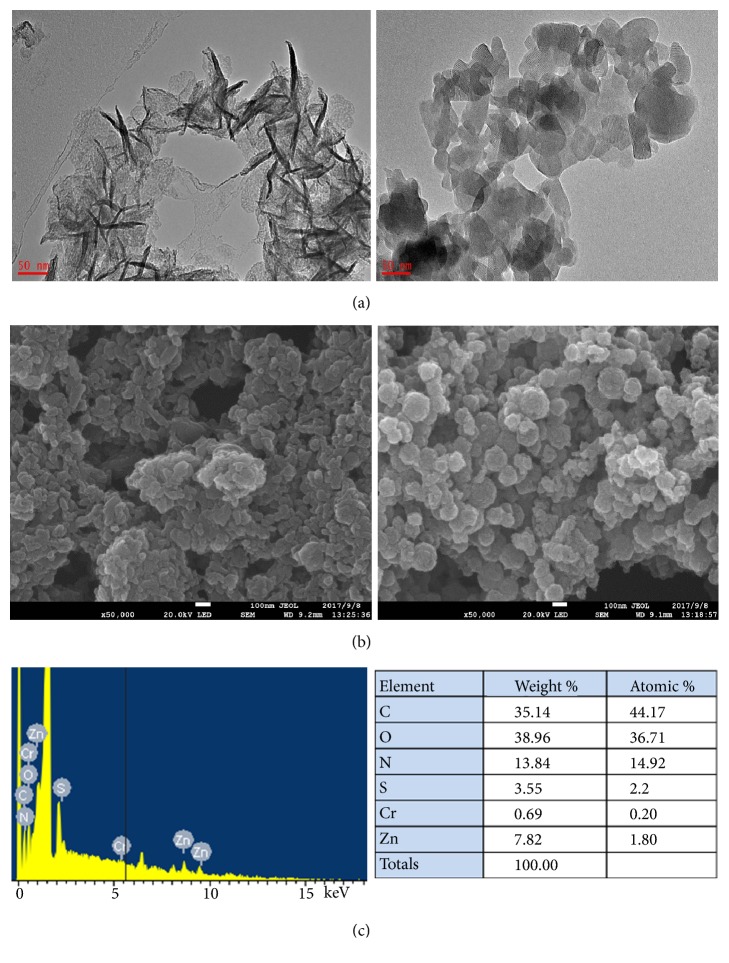
Characterization of the ZnCr LDH and ZnCr-ABTS LDH. TEM images of** (a) **ZnCr LDH (left) and ZnCr-ABTS LDH (right).** (b)** SEM images of ZnCr LDH (left) and ZnCr-ABTS LDH (right).** (c) **EDS point spectrum and relative corresponding elemental contents.

ABTS is an efficient redox mediator of laccase [24] and was therefore chosen for the LMS with laccase in this study. Laccases generally have optimal enzyme activity at an acidic pH (2.0–5.0) when ABTS is used as substrate [15]. Although the majority of LDHs are unstable in acidic solutions, there are a few exceptions, such as ZnCr LDH matrix [16]. Thus, this matrix was selected as the host matrix for ABTS in order to coordinate laccase activity at an acidic pH. ZnCr-ABTS LDH has been reported to be prepared by the direct coprecipitation method at pH5.0 [21]. The ZnCr LDH was light purple, whereas the as-synthesized ZnCr-ABTS LDH was light gray as shown in Figures 1(a) and 1(b). Notably, immediately after laccase addition, the suspension of ZnCr-ABTS LDH turned green, suggesting a redox reaction between ZnCr-ABTS LDH and laccase, because ABTS^+•^ was a stable and blue-green radical cation. Moreover, a green precipitate was observed after resting overnight, and the supernatant was colorless, suggesting that ABTS molecules had been successfully intercalated within the interlayer domain of ZnCr LDH rather than being present in solution. TEM images showed that the ZnCr LDH had a light sheet structure (Figure 2(a)). In contrast, ZnCr-ABTS LDH had a dark sheet structure, which could also be attributed to the green ABTS^+•^ in the interlayer domain of ZnCr LDH. Aggregation of the ZnCr LDH and ZnCr-ABTS LDH sheets was observed in the SEM images (Figure 2(b)). When compared with the original ZnCr LDH, no major morphological changes were observed for the LDH following ABTS intercalation. In addition, the chemical composition of the ZnCr-ABTS LDH determined by EDS showed that C, S, N, O, Cr, and Zn were present (Figure 2(c)), providing the best evidence for the formation of the ABTS LDH hybrid. Analysis of the elemental content in the matrix by EDS indicated an S content of 3.55 wt%, suggesting that the ABTS content was approximately 0.28 mmol/g in the solid LDH-ABTS sample.

**Figure 3:**
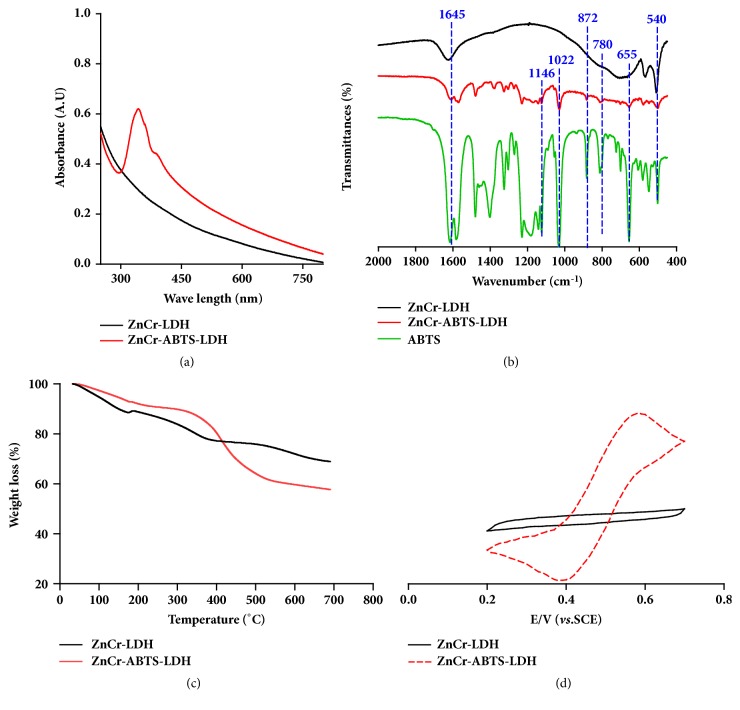
**(a) **UV-Vis absorption spectra.** (b)** Fourier transform-infrared spectra.** (c) **TGA thermograms.** (d)** Cyclic voltammograms of the ZnCr LDH and ZnCr-ABTS LDH modified GC electrode at a sweep rate of 20 mV/s in 0.05 M acetate buffer at pH 6.0.

For the UV-Vis absorption spectra of ZnCr LDH and ZnCr-ABTS LDH, two absorption peaks from the near-UV region were observed for LDH-ABTS (Figure 3(a)), which were attributed to the unsaturated double bonds of the ABTS molecules [25]. For the intercalated phase (ZnCr-ABTS), the major band at 340 nm should correspond to the intercalated ABTS dianion. The presence of the organic anions within the LDH interlayer domain was confirmed by FT-IR analysis (Figure 3(b)). The infrared spectrum of ZnCr-ABTS LDH showed bands at 1146, 1022, 872, and 655 cm^−1^, assigned to the stretching and bending modes of the sulfonate groups on ABTS, and bands at 1644, 780, and 540 cm^−1^, attributed to LDH [26]. The spectra of ZnCr-ABTS LDH contained the characteristic bands of both ABTS and LDH, confirming the formation of the ZnCr-ABTS LDH hybrid. The thermal stability of the ZnCr LDH and ZnCr-ABTS LDH hybrid was evaluated by TGA. As shown in Figure 3(c), the decomposition temperatures of ZnCr LDH and ZnCr-ABTS LDH were different from each other. The ZnCr-ABTS LDH hybrid showed decomposition in two steps. The first weight loss (115-200°C) indicated the loss of water from the samples. In comparison with the TGA curve of LDH, significant weight loss of about 20% was observed between 380°C and 530°C in the TGA curve of the ZnCr-ABTS LDH hybrid, corresponding to thermal degradation of ABTS from the ZnCr-ABTS LDH structure [27].

The results of these analyses confirmed that the LDH-ABTS hybrid was synthesized and that ABTS was firmly intercalated within the interlayer domain of LDH. This strong immobilization of the ABTS molecules within LDH could be explained by the following factors. First, the basal spacing of ZnCr LDH fit well with a monolayer arrangement of the ABTS^2-^ anion [28]. Additionally, owing to the high positive charge density of the LDH layers, the negatively charged ABTS^2-^ anion could be tightly attached to the LDH sheets by electronic interactions after its intercalation into the interlayer spacing of LDHs. These sufficiently strong ionic interactions could induce stable self-assembly of the two components [29]. Therefore, these factors prevented the rapid leaching of ABTS from the ZnCr-ABTS LDH hybrid in aqueous solution.

The electrochemical behavior of a GC coated by a film of as-synthesized LDHs at pH 6.0 in acetate buffer was examined by CV (Figure 3(d)). No signals were observed for LDH within a potential window between 0.2 and 0.7 V, but a well-defined reversible signal was observed at a mean peak potential of 0.49 V, which could be ascribed to the reversible oxidation of intercalated ABTS [27]. This peak potential was similar to that reported for ABTS in aqueous solution, and, indeed, in this electrochemical process, the ABTS underwent a reversible one-electron oxidation to a stable radical ABTS^+•^. This result confirmed that the electroactivity of ABTS was still maintained within the interlayer domain of LDHS. For the LDH-modified electrode, the electrochemical transfer proceeded by an intracrystalline mechanism, which was involved in electron hopping between electroactive cations such as Ni, Co, and Fe, present in the layer structure and motion of electrolyte anions. Furthermore, intercalated redox molecules, such as ABTS, can confer these LDH materials with specific electrochemical properties by improvement of interlayer electronic transfer [28]. Recently, these synthetic hybrid LDHs, such as ZnAl-ABTS LDH and CoAl-ABTS LDH, have been used as immobilization matrices for several oxidases, including laccase, horseradish peroxidase, and bilirubin oxidase, to facilitate the development of biosensors and biofuel cells [29].

### 3.2. Lac/ZnCr-ABTS LDH Encapsulation in Alginate

In this study, ZnCr LDH intercalated by ABTS was used as a matrix to adsorb laccase, resulting in Lac/ZnCr-ABTS LDH hybrids. Subsequently, the hybrids were entrapped into alginate beads, leading to coimmobilization of laccase and ABTS. Indeed, a powdered form of LDH may not be suitable in wastewater treatment systems because of the low hydraulic conductivity of the material and the resulting large sludge production [30]. In addition, most LDH materials are sensitive to acid conditions. Thus, entrapment of LDH materials into polysaccharides, such as chitosan, pectin, and alginate, has been widely applied with respect to water treatment since it is a simple and low-cost technique [31]. These alginate biohybrid beads can provide a protective coating that could improve the stability of LDHs at an acidic pH, promote the resistance of laccase to conformational changes in solution, and prevent the leaching of the intercalated redox molecules and the adsorbed enzymes from the LDH structure. More importantly, the as-prepared beads are more easily separated from aqueous solution than the powdered form of LDHs, contributing to the reusability of the LMS.

**Figure 4:**
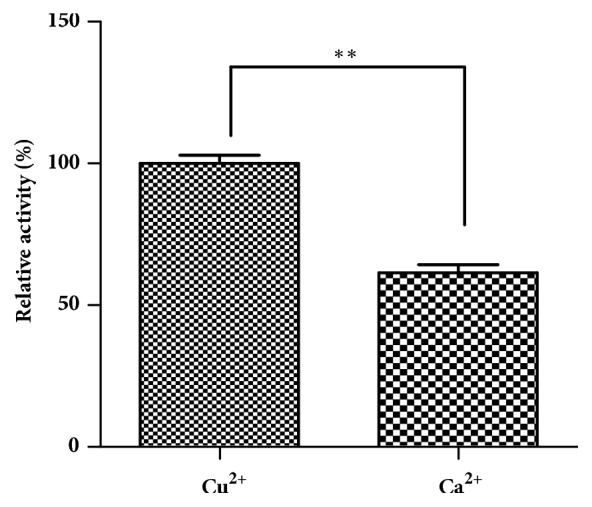
Relative activity of the Lac/ZnCr-ABTS LDH entrapped in alginate beads (3%) with different types of crosslinking agents.

**Figure 5:**
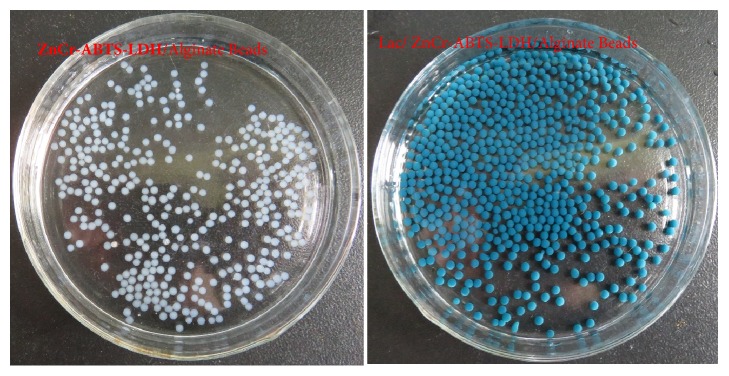
Photographic images of ZnCr-ABTS LDH/alginate beads (left) and Lac/ZnCr-ABTS LDH/alginate beads (right).

Under the same immobilization procedure, the relative activity of laccase in Lac/ZnCr-ABTS LDH/alginate beads (Im-LMS) was 61% when using the most common crosslinking agent (Figure 4). In contrast, the relative activity of laccase in Im-LMS was increased 1.6 times using Cu_2_SO_4_ as a crosslinking agent. About 87% of the laccase immobilization yield was obtained under the optimum conditions. The laccase activity was significantly higher when Lac/ZnCr-ABTS LDH was entrapped into Cu-alginate beads than when the material was entrapped into Ca-alginate beads. Because laccase is a copper-dependent enzyme and copper ions play important roles in the catalytic mechanism of laccase, enzymes immobilized in copper alginate tend to retain more activity [32]. Furthermore, there are clusters of histidine residues in laccase which provide additional high binding affinity sites for Cu(II) ion, leading to an increase in the adsorption capacity of Cu-alginate beads to laccase [33]. Thus, Cu-alginate beads were used for further experiments. The beads entrapping ZnCr-ABTS LDH were greyish white, while the beads entrapping Lac/ZnCr-ABTS LDH were bluish green with a diameter range of 3.0-5.0 mm in Figure 5.

### 3.3. Decolorization Capacity

**Figure 6:**
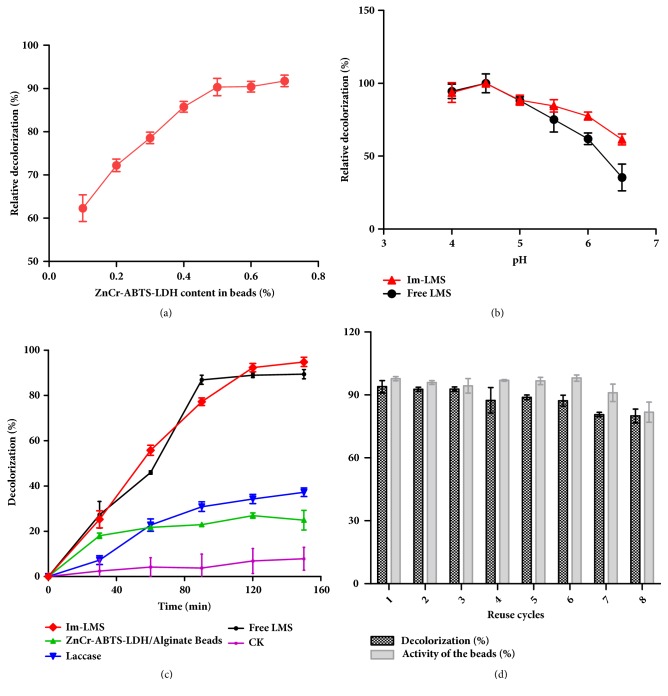
**(a)** Effects of ZnCr-ABTS LDH content in alginate beads on dye decolorization.** (b)** Effects of pH on dye decolorization by the free LMS (Lac/ABTS) and Im-LMS (Lac/ZnCr-ABTS LDH/alginate beads).** (c)** Dye decolorization by the free LMS, the Im-LMS, and laccase alone as a function of time. The decolorization conditions were as follows: 20 mg/L dye, 30 mg Im-LMS, or 1200 U/L laccase+2.1 mM ABTS or 1200 U/L laccase in 4.0 mL acetate buffer (pH 6.0).** (d) **Reusability of the Im-LMS (30 mg) over eight successive cycles using 20 mg/L dye in acetate buffer at pH 6.0, with shaking at 100 rpm for 2 h at 25°C.

The effects of the percentage of Lac/ZnCr-ABTS LDH content in Cu-alginate beads on dye decolorization are shown in Figure 6(a). The decolorization rate increased as the percentage of Lac/ZnCr-ABTS LDH in Cu-alginate beads increased. When the Lac/ZnCr-ABTS LDH content increased from 0.1% to 0.5%, the decolorization rate increased from 62.1% to 90.2%. However, there were no obvious differences in decolorization rates induced by 0.5% and 0.7% Lac/ZnCr-ABTS LDH /Cu-alginate beads. This result indicated that 0.5% Lac/ZnCr-ABTS LDH in Cu-alginate beads was effective for dye removal. Thus, for further experiments, 0.5% Lac/ZnCr-ABTS LDH in Cu-alginate beads was used. The effects of pH on the activities of free LMS (Lac/ABTS) and the Im-LMS were studied (Figure 6(b)). The optimum pH for maximum decolorization by the Im-LMS was 4.5, similar to that for free LMS, and this was attributed to the activity of laccase, which reached a maximum at pH 4.5 when ABTS was used as the substrate [12]. Increasing the pH further resulted in an evident decrease in the activities of both free LMS and immobilized LMS. However, the Im-LMS displayed higher activities than the free LMS beyond pH 4.5, indicating that the activity of LMS after being immobilized was less influenced by the environmental pH value. However, ZnCr LDH materials displayed increased instability as the pH decreased and showed slight degradation below pH 6.0. Because of the better stability and higher activity, acetate buffer at pH 6.0 was used for further dye decolorization by the Im-LMS.

The decolorization efficiency of the Im-LMS was examined via comparison of the decolorization efficiency of the free LMS, the Im-LMS, and laccase alone (Figure 6(c)). For ZnCr-ABTS/alginate beads without laccase, only ~20% of dye decolorization was achieved after 60 min, and this value remained almost constant as the reaction time increased. This removal was attributed to the adsorption of the dye molecules by the beads. For laccase alone, a low decolorization (~30%) was observed after 120 min, and decolorization slowly increased as the reaction time increased, indicating that the MG dye employed herein was not a typical laccase substrate and that a more efficient decolorization method would require a mediator. In addition, the MG in the control groups (without laccase) showed 10% decolorization at pH 6.0 in acetate buffer after 120 min of incubation. This decolorization was attributed to the chemical transformation of MG, in which chromatic MG reacted with hydroxide ions, resulting in a colorless leuco-form of MG at pH values greater than 4 [34, 35]. In contrast, almost complete removal (90%) was achieved within 90 min by the free LMS, and ~77% decolorization was achieved using the Im-LMS over the same period of time; for the latter, the decolorization rate reached 92% after 120 min. This result suggested that the Im-LMS exhibited a dye removal efficiency similar to that of the free LMS. The MG decolorization by laccase immobilized on various matrix was presented in the literature [36, 37]. For example, the decolorization yields of MG by laccase immobilized on titanium oxide were 90% within 6 h [36]. The decolorization yields of MG by cross-linked laccase aggregates were 95% within 6 h [37]. Moreover, the decolorization yields of MG by coimmobilized Laccase-acetylacetone system were 95% within 48 h [14]. In comparison, our result showed that Im-LMS exhibited a higher removal efficiency of MG.

The reuse and recycling of immobilized LMS for dye decolorization were examined over eight cycles, as shown in Figure 6(d). With a reaction time of 120 min, comparably high removal yields were obtained at every cycle using the recovered LMS. About 79% decolorization was observed, and more than 81% of enzyme activity was maintained after eight cycles, indicating that the removal efficiency and enzyme activity decreased gradually with increasing cycle number. This decline was mainly attributed to the accumulation of dye degradation products which depressed the activity of the enzyme and affected the reaction in the next cycle [13]. The other cause may be the loss of laccase and ABTS during the recycling and washing processes [22].

To date, several reports have described the reuse of LMS for dye decolorization. For example, an enzyme membrane reactor using a laccase/ABTS system was developed for continuous treatment of the dye solution [38]. During this process, there was an additional requirement to recover the mediator ABTS via ammonium sulfate precipitation. Moreover, the membrane reactor was also developed using TEMPO attached to PEG as mediator to explore the possibility of reusing LMS for azo dye decolorization [11]. Cost analysis of the processes demonstrated the feasibility of this system. However, for these methods, complex operations and expensive equipment may be major limitations in the application of LMS to bioremediation at an industrial scale. In this work, an immobilized LMS was fabricated by entrapping Lac/ZnCr-ABTS LDH into alginate beads, which is a simple and low-cost immobilization method. Moreover, the immobilized LMS could be utilized by mixing beads with dye effluents and could then be recovered by precipitation or filtration. Thus, the system was convenient for operating and controlling, and the process may be easily scaled up. Briefly, compared with the free LMS, the key advantage of the Im-LMS lies in its ability to be recycled easily, greatly reducing the running cost of the enzyme and mediator for practical applications.

### 3.4. Stability Analysis

**Figure 7:**
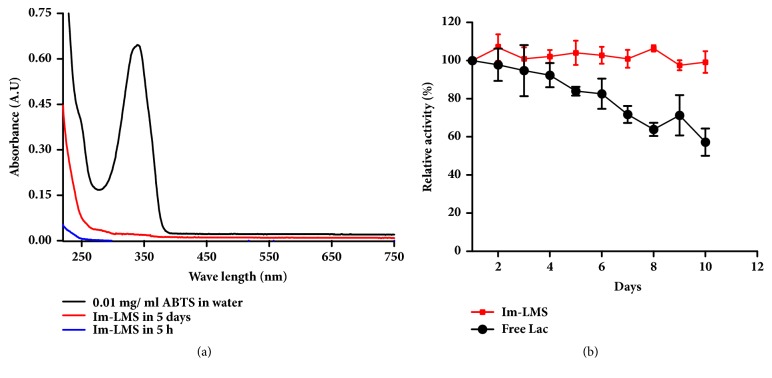
**(a) **UV-Vis spectra of the Im-LMS dispersed in water for 5 h or 5 days. The spectrum of ABTS is also shown for comparison.** (b) **Stability of the Im-LMS compared with that of the free laccase upon storage.

The possible leaching of ABTS from the immobilized LMS and its storage stability was then evaluated. Because ABTS has a characteristic UV-Vis absorption peak at ~340 nm, this signal was used to estimate the concentration of ABTS (Figure 7(a)). Hardly any signal assigned to ABTS was observed in the supernatant solution after 5 h, implying that the ABTS showed no leaching in the short term. Furthermore, a negligible signal was observed after an extended period of time (5 days), indicating that leaching of the ABTS was minimal. Notably, this weakness of the mediator (i.e., low leakage) could be negated by the reduced cost of the process due to reuse of the mediator. As shown in Figure 7(b), the activity of free laccase dropped much faster than that of the Im-LMS at the same storage conditions. The free laccase only retained ~50% of its original activity after 10 days. In comparison, the Im-LMS maintained about 100% of its original activity within the same time because the resistance of laccase to conformational changes in solution can be improved after immobilization [32]. These results confirmed the high stability of the Im-LMS.

### 3.5. Biodegradation of MG by the Im-LMS

**Figure 8:**
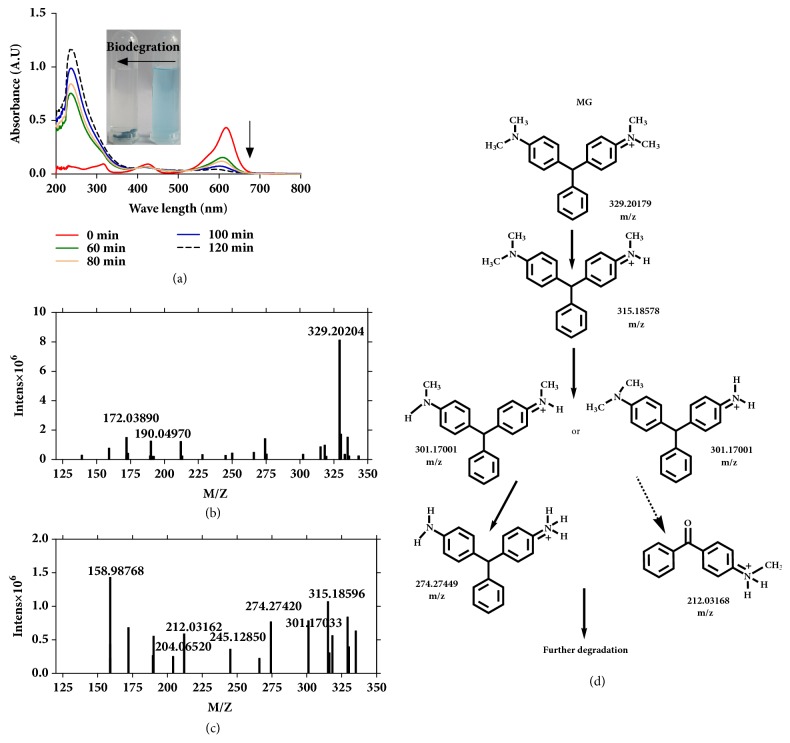
**(a) **UV-Vis spectra of the MG dye during the enzymatic degradation process at different time intervals. Also shown is a photograph of the sample both before and after decolorization. Positive-ion MALDI mass spectrum of MG.** (b)** Before enzymatic degradation.** (c)** Two hours after enzymatic degradation.** (d) **Proposed pathway of MG degradation after treatment with the Im-LMS.

There were obvious changes in the UV-Vis absorbance spectra of MG before and after dye decolorization as shown in Figure 8(a). For MG dye, three characteristic absorption peaks at 618, 424, and 320 nm were observed in the UV-Vis absorption spectra. The decolorization of MG dye solution was significant following treatment with the Im-LMS. This decolorization was also demonstrated by quantitative changes in the UV-Vis spectra. The characteristic absorption peaks (320, 424, and 618 nm) in MG decreased and even disappeared after reaction for 120 min. Furthermore, an obvious blue-shifted characteristic peak (618 nm) was observed as the interaction time increased. According to a previous study, successive N-demethylation of MG likely led to the blue shift in the *λ*max of the MG solution [39]. In addition, a sharp increase was observed in a new peak at 232 nm, suggesting that some new compounds with benzene rings had formed during the degradation process. To further identify the degradation products, MALDI-TOF-MS analysis was performed on the dye solution before and after treatment with the Im-LMS. Although MALDI-TOF-MS has generally been used to analyze relatively large molecules, such as proteins, this method has recently been used as an effective analytic tool for the analysis of phytochemicals/secondary metabolites and the identification of intermediate products formed during dye degradation [40, 41]. The MS spectrum of MG using *α*-cyano-4-hydroxycinnamic acid (*α*-CHCA) and sinapinic acid as the matrix before treatment is shown before treatment in Figure 8(b). A strong signal at* m/z* 329.20 corresponding to MG cations [39] and two peaks at* m/z* 172.03 and 190.04 assigned to *α*-CHCA were detected [40]. After a decolorization time of 120 min, the peak at* m/z* 329.20 declined sharply, and new peaks appeared at* m/z* 315.18, 301.17, 274.27, 245.12, 204.06, 212.03, and 158.98 (Figure 8(c)). The degradation products at* m/z* 315.18, 301.17, and 274.27 are likely to represent the N-demethylation-dependent oxidative degradation of MG [42]. Other new peaks at* m/z* 245.12, 212.03, 204.06, and 158.98 implied that the further breakdown of the aromatic ring occurred in the decolorization process.

Based on the results of MALDI-TOF-MS analysis, Figure 8(d) shows the probable bond-breaking and structures of the intermediates. Based on these findings, we were able to propose a degradation pathway for MG. We concluded that the N-demethylation of MG proceeded upon decolorization by the Im-LMS, resulting in successive intermediates, such as desmethyl MG, didesmethyl MG, and tridesmethyl MG, which contributed to the decreases in characteristic absorption peaks (424 and 618 nm) and the blue shift in the visible region. Subsequently, the aromatic ring in MG was gradually destroyed, and this process was relatively slow in comparison with the N-demethylation. Finally, these intermediates further broke down into small molecules with one benzene ring.

### 3.6. Toxicity Analysis

**Figure 9:**
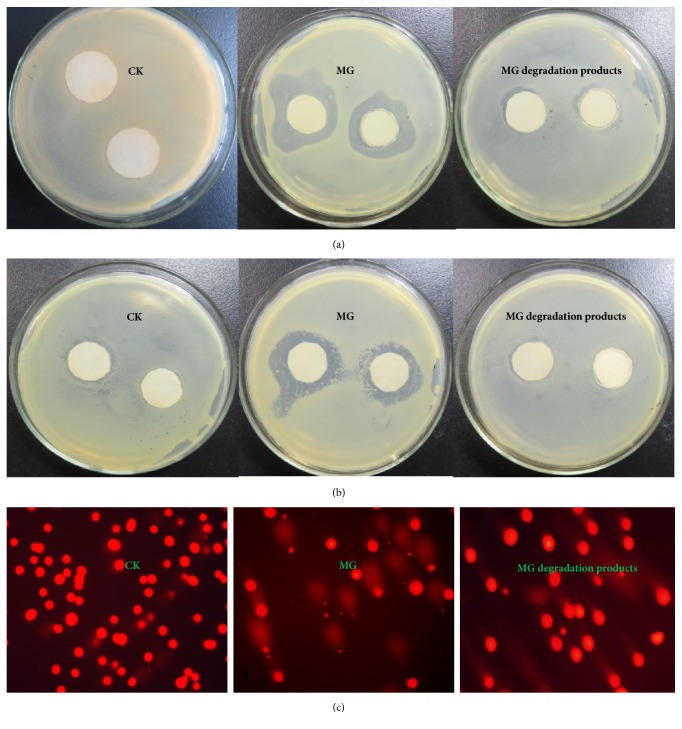
(a) Inhibition zone of* E. coli* CICC 23872. (b) Inhibition zone of S. aureus CICC 23926. (c) Representative comet images showing different levels of damage to HepG2 cells treated with MG and its degradation product.

**Figure 10:**
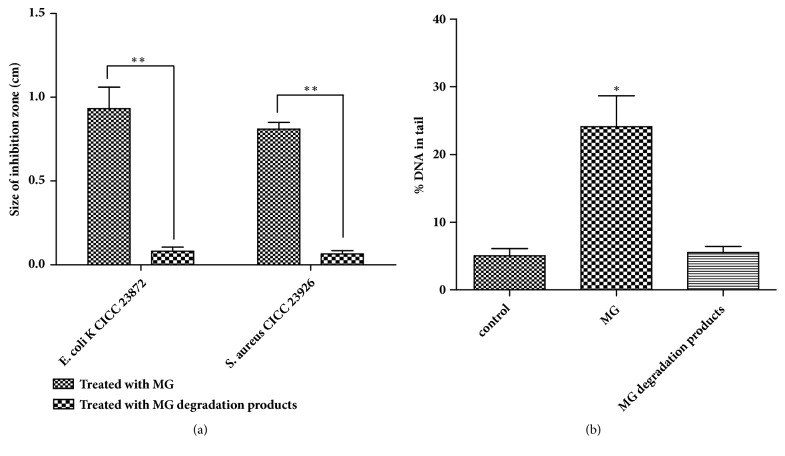
Toxicity analysis of MG and its degradation products.** (a)** The sizes of the inhibition zones of* E. coli *CICC 23872 and* S. aureus* CICC 23926.** (b) **Comet assays.

Trace amounts of MG in the environment can result in high toxicity to cells of animals, plants, and microorganisms [43, 44]. Thus, MG is considered a significant risk to environmental, ecosystem, and human health. To determine whether the toxicity of MG degradation products is reduced, the toxicity of MG solution before and after treatment was examined using antimicrobial activity tests and genotoxicity assays. A notable inhibition zone for both* E. coli *CICC 23872 and* S. aureus *CICC 23926 was observed when MG was added to the paper disks (Figures 9(a) and 9(b)). However, the inhibition zone became significantly smaller when MG degradation products were added; however, this difference was not significant (*p* > 0.05), suggesting that MG degradation products were less toxic than MG (Figure 10(a)).

Comet assays are highly sensitive tools for the detection of genotoxic compounds [23]. HepG2 cells are derived from human liver hepatocellular carcinoma and are considered a relevant model for human exposure. The normal cells had an intact nucleus and no comet tail. However, most cells exposed to MG showed heavy damage, and their nuclei became small or disappeared, resulting in the occurrence of strong fluorescence signals in comet tails (Figure 9(c)). In contrast, few cells exposed to MG degradation products were slightly damaged, and fluorescence signals in comet tails were weak because the nuclei remained intact. In addition, compared with that in cells exposed to MG, the percent DNA in the comet tail in cells exposed to MG degradation products decreased sharply (Figure 10(b)). There were no significant differences between cells exposed to MG degradation products and control cells. Thus, the MG dye after treatment with Im-LMS was markedly detoxified. Previous studies have shown that the toxicity of the dye was not completely reduced, despite nearly complete decolorization by free LMS [45]. This was partly attributed to the addition of mediator compounds, such as HOBT, which have some toxic effects. However, our results suggested that the use of the Im-LMS may help to overcome this problem.

## 4. Conclusion

For practical applications using the LMS, the high costs of enzymes, and mediators and the potential toxicities of dissolved mediators are major limitations. In this study, we proposed a simple procedure to coimmobilize laccase and ABTS in LDH/alginate biohybrid beads. The coimmobilized laccase-ABTS system (Im-LMS) favored the reuse of mediators and enzymes and reduced the toxicity of the mediator. Our findings suggested that the MG dye could be effectively degraded by Im-LMS and that the toxicity of MG after treatment was reduced. The recyclability and stability of Im-LMS may reduce the using cost of LMS in dye effluent treatment.

## Data Availability

The data used to support the findings of this study are available from the corresponding author upon request.
